# Renal involvement and metabolic alterations in adults patients affected by cystic fibrosis

**DOI:** 10.1186/s12967-019-02139-4

**Published:** 2019-11-25

**Authors:** Silvia Lai, Sandro Mazzaferro, Anna Paola Mitterhofer, Enea Bonci, Paolo Giangreco Marotta, Francesco Pelligra, Manuel Murciano, Camilla Celani, Patrizia Troiani, Giuseppe Cimino, Paolo Palange

**Affiliations:** 1 Department of Translational and Precision Medicine, Nephrology and Dialysis Unit, “Sapienza” University of Rome, Viale dell’Università 37, 00185 Rome, Italy; 2 Department of Experimental Medicine, “Sapienza” University of Rome, Rome, Italy; 3 Department of Public Health and Infectious Diseases, Special Unit of Cystic Fibrosis, “Sapienza” University of Rome, Rome, Italy; 4 Department of Specialist Pediatric, Rheumatology Unit, Bambino Gesù Children’s Hospital, Rome, Italy

**Keywords:** Cystic fibrosis, Chronic kidney disease, Cardiovascular disease, Endothelial dysfunction

## Abstract

**Background:**

Cystic fibrosis (CF) is one of the most frequent genetic diseases and the median survival of these patients has improved in the last few decades, therefore it becomes necessary to evaluate the long-term complications as renal and cardiovascular risk factors.

**Aim of the study:**

To evaluate the incidence, the manifestations of renal disease and the possible association with metabolic and endothelial dysfunction markers in the CF population.

**Materials and methods:**

We performed a cross-sectional, observational study on 226 CF patients. Clinical and laboratory instrumental parameters (metabolic, inflammatory and endothelial dysfunction markers) were evaluated.

**Results:**

We showed 65 patients with chronic kidney disease (CKD) and 158 patients with a reduced value of forced expiratory volume in 1 s (FEV1), of which 58 patients with a severe reduction of FEV1. Moreover 28 patients had undergone lung transplantation and them had a significant lower estimated Glomerular Filtration Rate (eGFR) with respect to the non-transplanted patients (p < 0.001). We reported also a significant association between lower eGFR value and serum triglycerides, total cholesterol and low-density lipoproteins (LDL) (p = 0.005, p < 0.001, p = 0.040; respectively), with a significant negative correlation between eGFR and serum triglycerides (r = − 0.28; p < 0.01). Moreover we found a significant association between lower eGFR value and serum uric acid (SUA) (p = 0.005), while we did not found an association with 25-hydroxy-vitamin-D value, serum glucose and hemoglobin A1c levels.

**Conclusions:**

Our study showed a high prevalence of CKD in CF patients. Moreover we showed an increase of endothelial dysfunction and metabolic indexes in patients with reduced renal function, as SUA, serum triglycerides and LDL, suggesting the need for an early and complete screening of the main metabolic indexes to reduce cardiovascular risk and progression of renal damage, in particular in patients with lung transplant.

## Background

Cystic fibrosis (CF) is an autosomal recessive genetic disease, with a high prevalence in Europe, North America and Australia [1, 2], involving about between 1:2500 and 1:1800 people and it is therefore classified as one of the most frequent genetic diseases. The disease is caused by the mutation of the Cystic Fibrosis Transmembrane Regulator (CFTR) gene [1, 2] that maps on chromosome 7 and encodes the CFTR protein, which is a transmembrane channel of chlorine [3, 4]. This channel regulates the transport of anions and ciliary mucus clearance in lungs, its dysfunction then leads to mucosal retention, chronic pulmonary infections, inflammation and progressively to lung damage with respiratory failure [5, 6]. Cystic fibrosis is a systemic disease that affects the epithelial cells of the pancreas (85–90% of cases) causing malabsorption and pancreatic insufficiency [7–9], the liver with biliary cirrhosis, male genital system causing atrophy and agenesis of the deferent ducts, then infertility [10, 11]. The increasing longevity among CF patients exposes them to the risk of developing nephropathy, this disease is known as CF related kidney disease (CFKD) [12, 13]. Renal involvement in CF may be associated with the underlying disease, with the comorbidities and with the treatment that patients receive. In fact the CFTR gene is expressed also in the kidneys, causing alterations in electrolyte homeostasis, but until now renal involvement has been little studied. In addition, comorbidities such as diabetes could cause nephropathy. However the most studied and known cause of renal injury in CF patients is iatrogenic, related to the intake of high doses of aminoglycosides (3–5 mg/kg/dose) during pulmonary exacerbations [14, 15]. Aminoglycosides are characterized by renal excretion through glomerular filtration, up to 15% is reabsorbed into the proximal tubule through a saturable mechanism, once overcome phenomena of cellular apoptosis occur, therefore presents a dose-dependent action. In the treatment of patients with CF the therapeutic dose is comparable to the toxic dose [16–19]. With advances in the therapy of CF, life expectancy has increased, and some previously unobserved disease associations are now seen in patients with CF. The aim of this study is to evaluate the incidence, the manifestations of renal disease and the possible association with metabolic and endothelial dysfunction markers in the CF population.

## Materials and methods

The study protocol was approved by the Clinical Research Ethics Committee of Sapienza, University of Rome, Italy. The study conforms to the principles outlined in the Declaration of Helsinki and we obtained a written consent by each patient enrolled.

### Study design and subjects

We performed a cross-sectional, single-center, observational study, without control group, that includes patients aged at least 18 years with diagnosis of CF, afferent to the CF center at the University Hospital “Policlinico Umberto I” of Rome, Sapienza University of Rome, Italy. Prospective data collection for each subject occurred within a 3–6 months time period and included clinical, laboratory and instrumental parameters.

### Patients

A total of 226 patients (126 males) were evaluated. The eGFR was evaluated according to the modification of diet in renal disease formula (MDRD), CKD-Epidemiology [16]. The state of arterial hypertension was defined by use of hypotensive drugs (angiotensin converting enzyme, angiotensin II receptor blockers, beta-blockers, calcium antagonists, alpha-lytic and/or diuretics) or by the presence of a pressure higher than 140/85 mmHg in three consecutive measurements.

### Inclusion criteria

Patients aged at least 18 years with diagnosis of CF.

### Exclusion criteria

We excluded patients affected by heart failure, psychiatric disorder, neoplastic diseases, and acute coronary syndrome within 3 months before the study. Moreover, patients that refused to give consent and patients with missing data were also excluded.

### Laboratory measurements

Blood was drawn in the morning after an overnight fasting of at least 12 h.

Standard automated techniques have been used to analyze the samples of all patients as follows: plasma glucose (mmol/L), hemoglobin A1c (HbA1c)(%), total serum cholesterol (mg/dL), triglycerides (mg/dL), high-density lipoprotein (mg/dL), creatinine (mg/dL), serum nitrogen (mg/dL), serum uric acid (SUA) (mmol/L), calcium (mg/dL), serum electrolytes (mEq/L), C-reactive protein (μg/L). Low-density lipoprotein cholesterol was calculated using the Friedewald equation: low-density lipoprotein (LDL) (mg/dL) = total cholesterol − high-density lipoprotein − (triglycerides/5).

25-hydroxy-vitamin-D (25-OH-VitD) (ng/mL) was measured by radioimmunoassay. Serum albumin (g/dL) was determined by bromcresol purple method.

### Diagnosis of cystic fibrosis

Immunoreactive trypsin test—IRT in neonatal age was performed. The genetic variants of CFTR were analysed by sequencing analysis and multiple ligation-dependent probe amplification (MLPA) if necessary 17/19 and IR/Del sequencing were performed (INNO-LiPA^®^ CFTR19 (20T)). If the mutation is not among the most common has been necessary to carry out molecular investigation of the whole gene for identifying exon deletions.

### Blood pressure measurements

Clinic blood pressure measurements have been performed by a standard automatic sphygmomanometer according to the British Hypertension Society guidelines [20]. Then, the mean values for systolic blood pressure and diastolic blood pressure were calculated for all participants. Hypertension was defined as SBP ≥ 140 mmHg or DBP ≥ 80 mmHg on repeated measurements.

### Respiratory function testing (Spirometry)

Spirometries were executed with the subjects in sitting position, wearing a nose clip according to the international guidelines [21, 22]. Before testing each subject, the spirometer was calibrated using a certified 3-L syringe. A laboratory spirometer and Quark spirometry software (Quark PFT Suite Version 9.1a, COSMED, Pavona, Italy) were used to measure forced expiratory volume in 1 s (FEV1) and forced vital capacity (FVC).

### Statistical analysis

Data management and analysis were performed using IBM^®^ SPSS^®^ Statistics 22.0 for Windows^®^ software (IBM Corporation, New Orchard Road Armonk, New York, United States). The normality of variables was tested using the Shapiro–Wilk method for normal distributions. Continuous variables (i.e.: age, eGFR, respiratory function parameters, laboratory parameters) were expressed as average ± standard deviation; categorical variables (e.g.: absolute values, gender, genetic mutations, transplant undergone) were expressed as percentage. Some variables such as eGRF, FEV1, and creatinine were recorded into categories based on their clinical significance. The hypothesis testing was performed using univariate analysis. Using the following tests: Chi squared test (χ^2^), t-Student, analysis of variance (ANOVA) and Bivariate Correlation (Pearson’s *r*) when each test was appropriate to use; values of p < 0.05 were considered statistically significant.

## Results

**Fig. 1:**
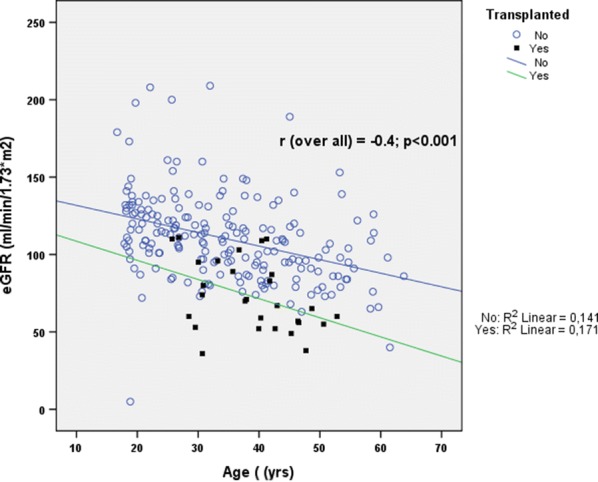
eGFR report and age in transplanted and non-transplanted patients. *eGFR* estimated Glomerular Filtration Rate

**Fig. 2:**
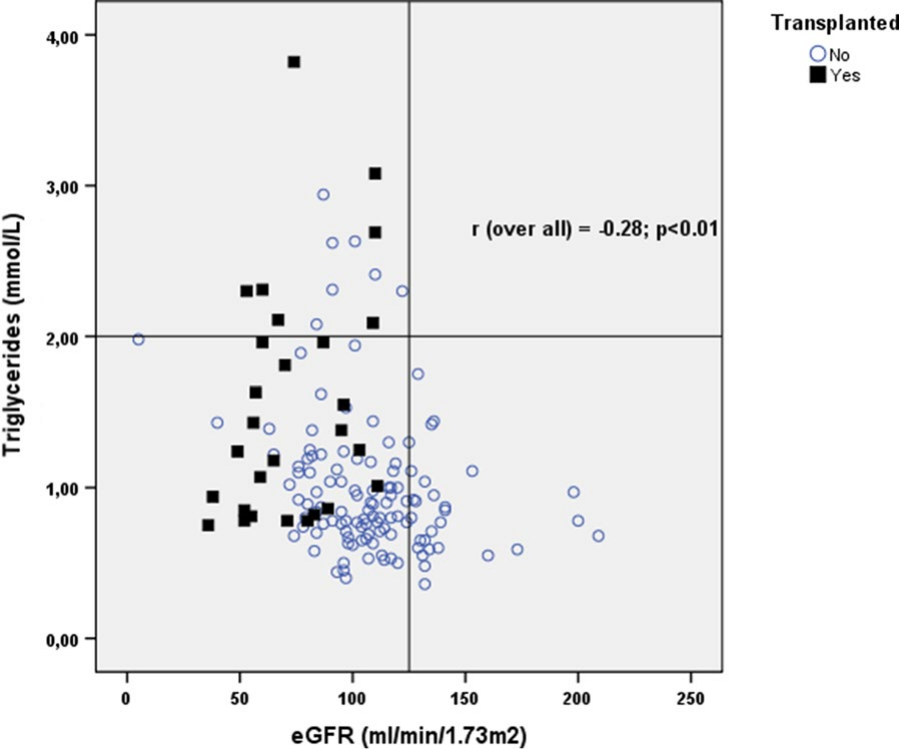
Relationship triglycerides and eGFR in transplanted and non-transplanted patients. *eGFR* estimated Glomerular Filtration Rate

Patient’s characteristics are shown in Table 1. A total of 226 patients (126 males) with the average age of 34.92 ± 11.67 years were evaluated. We divided the population into three groups evaluating the eGFR and the FEV 1 (Tables 2, 3) and we showed that 65 patients (28.8%) (28 males) have a reduced value of eGFR < 90 mL/min/1.73 m^2^ and 158 patients have a reduced value of FEV1, of which 58 patients (26%) (34 males) with a severe reduction of FEV1. Moreover we have stratified the population by the CFTR mutation classes and eGFR, confirming the prevalence of mutation class II with a major number of patients with reduced eGFR (Table 4). On a total of 226 patients, 28 had undergone lung transplantation, and we found that these patients had a significant lower eGFR with respect to the non-transplanted patients (p < 0.001) (Table 5), while we did not shown a significant difference in FEV1. Moreover we showed that eGFR and age are inversely proportional, but eGFR is significant lower in transplant patients compared to non-transplanted patients of the same age (p < 0.001) (Fig. 1). We reported also a significant association between lower eGFR value and serum triglyceride, total cholesterol and LDL (p = 0.005, p < 0.001, p = 0.040; respectively) (Table 6), with a significant negative correlation between eGFR and serum triglyceride (Fig. 2). Moreover we found a significant association between lower eGFR value and SUA (p = 0.005), while we did not found an association with 25-OH-VitD value, serum glucose and HbA1c (Table 6).

**Table 1:** Patients’ characteristics

	Age (years)	eGFR mL/min/1.73 m^2^	FEV_1_ %	Creatinine mg/dL
Males N = 126	34.8 ± 11.7	107.0 ± 28.5	71.6 ± 28.9	0.87 ± 0.21
Females N = 100	35.2 ± 11.7	104.1 ± 32.1	71.2 ± 24.9	0.71 ± 0.19
Total N = 226	34.92 ± 11.67	105.76 ± 30.08	71.41 ± 27.11	0.80 ± 0.22
p value (using t test)	0.909	0.477	0.923	0.001

Comparison of the mean age, eGFR, FEV1 and creatinine stratified for sex. Data are shown as Average ± SD

*FEV1* forced expiratory volume in the first second, *eGFR* estimated Glomerular Filtration Rate

**Table 2:** Patients’ characteristics

	eGFR < 90 mL/min/1.73 m^2^	eGFR 90–119 mL/min/1.73 m^2^	eGFR ≥ 120 mL/min/1.73 m^2^	Total
Males N = 126	22.2% (28 pts)	50% (63 pts)	27.8% (35 pts)	126
Females N = 100	37% (37 pts)	34% (34 pts)	29% (29 pts)	100
Total N = 226	28.8% (65 pts)	42,9% (97 pts)	28.3% (64 pts)	226
p value (using Chi squared test (χ^2^ 2df)				0.023

Mean eGFR comparison between sex stratified groups. Data are shown as Average ± SD

*eGFR* estimated Glomerular Filtration Rate, *pts* patients

**Table 3:** Patients’ characteristics

	FEV1 ≤ 50%	FEV1 51–90%	FEV1 > 90%	Total
Males N = 126	27,2% (34 pts)	41,6% (52 pts)	31,2% (39 pts)	125
Females N = 100	24,5% (24 pts)	49% (48 pts)	26,5% (26 pts)	98
Total N = 226	26% (58 pts)	44,8% (100 pts)	29,1% (65 pts)	223
p value (using Chi squared test (χ^2^ 2df)				0.540

Mean FEV1 comparison between sex stratified groups. Data are shown as Average ± SD

*FEV1* forced expiratory volume in the first second, *pts* patients

**Table 4:** Patients’ characteristics

	eGFR < 90 mL/min/1,73 m^2^	eGFR 90–119 mL/min/1.73 m^2^	eGFR > 120 mL/min/1.73 m^2^	Total
Mutationclass I	25% (7 pts)	39.3% (11 pts)	35.7% (10 pts)	28
Mutationclass II	24.7% (39 pts)	45.6% (72 pts)	29.7% (47 pts)	158
Mutationclass III	0	66.7% (2 pts)	33.3% (1 pts)	3
Mutationclass IV	62.5% (5 pts)	25% (2 pts)	12.5% (1 pts)	8
Mutationclass V	33.3% (4 pts)	41.7% (5 pts)	25% (3 pts)	12
Total	26.3% (55 pts)	44% (92 pts)	29.7% (62 pts)	209
p value (using Chi squared test (χ^2^ 8df)				0.467

Data are shown as Average ± SD; Stratification into three groups according to eGFR for each CFTR mutation class

*eGFR* estimated Glomerular Filtration Rate, *pts* patients

**Table 5:** eGFR and FEV1 in transplanted and non-transplanted CF patients

	Non-transplanted patients N = 198	Transplanted patients N = 28	p value (using t-test)
eGFR (mL/min/1.73 m^2^)	110.38 ± 28.1	73.11 ± 22.7	0.001
FEV_1_ (%)	71.34 ± 27.1	71.89 ± 27.3	0.920

Data are show as Average ± SD

*eGFR* estimated Glomerular Filtration Rate, *FEV1* forced expiratory volume in the first second, *CF* cystic fibrosis

**Table 6:** Patients’ characteristics

eGFR	Age (years)	SUA (mmol/L)	25-OH-VitD (ng/mL)	Glycemia (mmol/L)	HbA1c (%)	Triglycerides (mmol/L)	Total cholesterol (mmol/L)	LDL (mmol/L)
< 90 mL/min/1.73 m^2^	41.64 ± 10.39	0.38 ± 0.10	26.85 ± 13.23	5.29 ± 1.31	5.98 ± 1.02	1.31 ± 0.64	4.50 ± 1.05	2.21 ± 0.92
90–119 mL/min/1.73 m^2^	34.63 ± 10.80	0.34 ± 0.08	25.55 ± 9.70	5.41 ± 2.09	5.69 ± 0.93	1.07 ± 0.60	3.90 ± 1.02	1.97 ± 0.79
≥ 120 mL/min/1.73 m^2^	28.54 ± 10.52	0.32 ± 0.09	21.96 ± 7.80	5.29 ± 1.31	5.98 ± 1.02	0.89 ± 0.39	3.41 ± 1.13	1.72 ± 0.89
p value (using ANOVA)	0.001	0.005	0.197	0.671	0.146	0.005	0.001	0.040

Lipid and Metabolic profile in relation to eGFR and age in CF patients. Data are shown as Average ± SD

*CF* cystic fibrosis, *eGFR* estimated Glomerular Filtration Rate, *LDL* low-density, *SUA* Serum Uric Acid, *25-OH-VitD* 25-hydroxy-vitamin D, *HbA1c* hemoglobin *A1c*. lipoprotein cholesterol, *CF* cystic fibrosis

## Discussion

More than 2000 mutations of CFTR gene have been identified, the most frequent variation is ΔF508 (Class II), characterized by the deletion of three nucleotides with the loss of the codon coding for phenylalanine [4, 6]. This mutation involves the altered formation of the CFTR protein that is degraded by proteasomes in the endoplasmic reticulum, with a lack of exposure of the channel on the membrane. The various mutations are categorized into six classes [3] depending on the type of protein deficit which has been found. The effects of CF are not limited to the lung alone, although, the major cause of mortality is associated with bronchiectasis and subsequent infections. Early diagnosis, intensive antibiotic therapy and developments in supportive treatment have extended the life expectancy for CF patients by reducing mortality and morbidity. Due to the increase in patient lifespans, previously unobserved disease associations have been detected. Renal involvement in CF patients has always been considered rare, but recently, renal pathologies such as glomerulosclerosis, mesangial proliferation, membranoproliferative and postinfectious glomerulonephritis, nephrocalcinosis and hematuria, tubular damage, fibrillary glomerulonephritis, and amyloidosis, in particular amyloid protein A (AA) in children, have been reported [23]. There has been a dramatic increase in median survival of CF patients over the last two decades, in fact median survival has improved to 45 years, therefore it becomes necessary to deal with long-term complications such as renal and cardiovascular diseases. Acute kidney injury (AKI) in patients with CF is well documented in association with episodes of infection and use of antibiotics [18], while the prevalence and possible causes of CKD remains more debated. In our study we showed a prevalence of CKD of 28.8%, while another study [19] reported a prevalence of 14.2% of CKD with the same prevalent mutation (∆F508, class II). Moreover, in our study, we showed a reduced eGFR in lung transplanted patients with respect to the non-transplanted patients. Also Degen et al. [24] showed a worse renal function in transplanted patients, probably due to the use of immunosuppressive drugs such as cyclosporine and other calcineurin inhibitors. We reported also an increase of metabolic indexes, as triglycerides, total cholesterol and LDL, progression factors of renal damage and cardiovascular risk factors. The increase of serum triglycerides in patients with CKD is due to a dual mechanism, increased synthesis and reduced clearance [25], determining a lipid profile that favors atherosclerosis and consequent increase in cardiovascular risk. Moreover, we found an increase of SUA in patients with worse renal function. In recent years it has been shown that high levels of SUA are associated with renal and cardiovascular events, mainly due to renal glomerular vasoconstriction [26]. In a 7-year follow-up study of a sample of 177.570 individuals, patients with high SUA were found to have a 26% increased risk of developing CKD [27]. Hyperuricemia is involved in endothelial dysfunction by increasing inflammation and oxidative stress, however is unclear whether it is an individual risk factor or if it is associated with other risk factors such as hypertension, metabolic syndrome and CKD [28]. The integrity of the endothelium plays an important role in the maintenance of homeostasis by regulating the balance between vasoconstriction and vasodilation, in fact a possible mechanism of dysfunction related to SUA is given by the decoupling of xanthine-oxido-reductase and endothelial nitric-oxide-synthase (eNOS). Xanthine-oxido-reductase catalyzes the oxidative reaction of hypoxanthine in xanthine and finally in urate, in the metabolic process of purines. Xanthine-oxido-reductase exists in two forms, xanthine-dehydrogenase (XD) and xanthine-oxidase (XO), the former uses as NAD^+^ acceptor and NADH will be formed at the end of the conversion process. The XO instead uses molecular oxygen as an electron acceptor, at the end of the process anion peroxide and hydrogen peroxide will form. In a condition in which the XO is misused therefore, not only SUA, but also reactive oxygen species will have a deleterious effect on the endothelial component [29, 30]. According to the literature, 20% of adolescents and 40–50% of adults have CF related diabetes (CFRD), distinct from type 1 or type 2 diabetes [11]. In our study we showed higher levels of glycemia and HbA1c in patients with reduced eGFR even if not statistically significant. Cystic fibrosis related diabetes is the end-point of a spectrum of glucose abnormalities in CF that begins with early insulin deficiency and is associated with accelerated nutritional decline and deterioration of lung function [31]. Microvascular complications can occur, but the main cause of death is respiratory failure rather than cardiovascular causes as in type 1 or type 2 diabetes [32]. In our study we showed a reduced value of FEV1 in 158 patients (70.8%) even if we did not shown significant difference in patients with CKD and between transplanted and non-transplanted patients. Pulmonary function tests can yield measurements of lung capacity, forced expiratory flow, vital capacity and residual volume, but one of the most important spirometric parameter in CF is FEV1, an index of airway obstruction, that plays an important role in both clinical care and research [33]. The strong relationship between FEV1 and the pathophysiology of this chronic respiratory disease, combined with the ability to be objectively and reliably measured relatively to other endpoints, has made FEV1 a key endpoint to measure both efficacy and safety in CF clinical trials. There has been a dramatic increase in median survival of these patients in the last few years, but respiratory failure remains the leading cause of death among CF patients, and FEV1 remains an established marker of disease progression that can be used to evaluate the clinical course and therapeutic efficacy [33].

## Limitations

The limitations of our study are the limited sample size of CF patients, and the cross-sectional, single center study without control group. The limitation for all single-centre analysis is the potential lack of generalizability. Additional prospective follow-up studies with a larger number of patients are necessary to confirm our results.

## Conclusions

Our study shows a high prevalence of CKD in CF patients. Moreover we showed an increase of endothelial dysfunction and dyslipidemia indexes in patients with reduced renal function, as SUA, serum triglycerides and LDL, suggesting the need for an early and complete screening of the main metabolic indexes to reduce cardiovascular risk and progression of renal damage. Furthermore, a careful and frequent evaluation of eGFR could be an important predictor and control factor for renal damage, especially in patients with lung transplantation.

## Data Availability

The datasets generated during and/or analysed during the current study are available from the corresponding author on reasonable request.

## Abbreviations

25-OH-VitD: 25-hydroxy-vitamin-D
CF: cystic fibrosis
CFKD: CF related kidney disease
CFRD: CF related diabetes
CFTR: Cystic Fibrosis Transmembrane Regulator
CKD: chronic kidney disease
eGFR: estimated Glomerular Filtration Rate
eNOS: endothelial nitric-oxide-synthase
FEV1: forced expiratory volume in 1 s
FVC: forced vital capacity
HbA1c: hemoglobin A1c
LDL: low-density lipoproteins
MDRD: modification of diet in renal disease formula
SUA: serum uric acid
XD: xanthine-dehydrogenase
XO: xanthine-oxidase
